# Supplementary material for: Ultrafast dynamics of heme distortion in the O2-sensor of a thermophilic anaerobe bacterium
Source: Commun Chem. 2021 Mar 5;4:31. doi: 10.1038/s42004-021-00471-9 (PMC9814294; doi:10.1038/s42004-021-00471-9)
Supplement: Supplementary file 1 — Supplementary Information [file 42004_2021_471_MOESM1_ESM.pdf]

Ultrafast dynamics of heme distortion in the O_2-sensor of a thermophilic anaerobe bacterium

Olga N. Petrova[1,5], Byung-Kuk Yoo[1,6], Isabelle Lamarre[1], Julien Selles[2], Pierre Nioche[3,4] & Michel Negrerie[1,*]

[1]Laboratoire d'Optique et Biosciences, INSERM U1182, Ecole Polytechnique, 91120 Palaiseau, France.
[2]Laboratoire de Biologie du Chloroplaste et Perception de la Lumière chez les Micro-Algues, UMR 7141 CNRS-Sorbonne Université, Institut de Biologie Physico-Chimique, 75005 Paris, France.
[3]Laboratoire de Toxicité Environmentale, Cibles Thérapeutiques, Signalisation Cellulaire et Biomarqueurs, UMR S1124, Campus Saint-Germain-des-Prés, Université de Paris, 75006 Paris, France.
[4]Plateforme d'Analyses Moléculaires et Structurales, BioMedTech Facilities, INSERM US36 - CNRS UMS2009, Campus Saint-Germain-des-Prés, Université de Paris, 75006, Paris, France.
[5]Present address: O.N.P. Laboratoire Handicap Neuromusculaire: Physiologie, Biothérapie et Pharmacologie Appliquées, Inserm U1179, Université de Versailles Saint-Quentin-en-Yvelines, Montigny-le-Bretonneux, France.
[6]Present address: Department of Chemistry and Chemical Engineering, California Institute of Technology, Pasadena, CA 91125, USA.

Email: michel.negrerie@polytechnique.edu

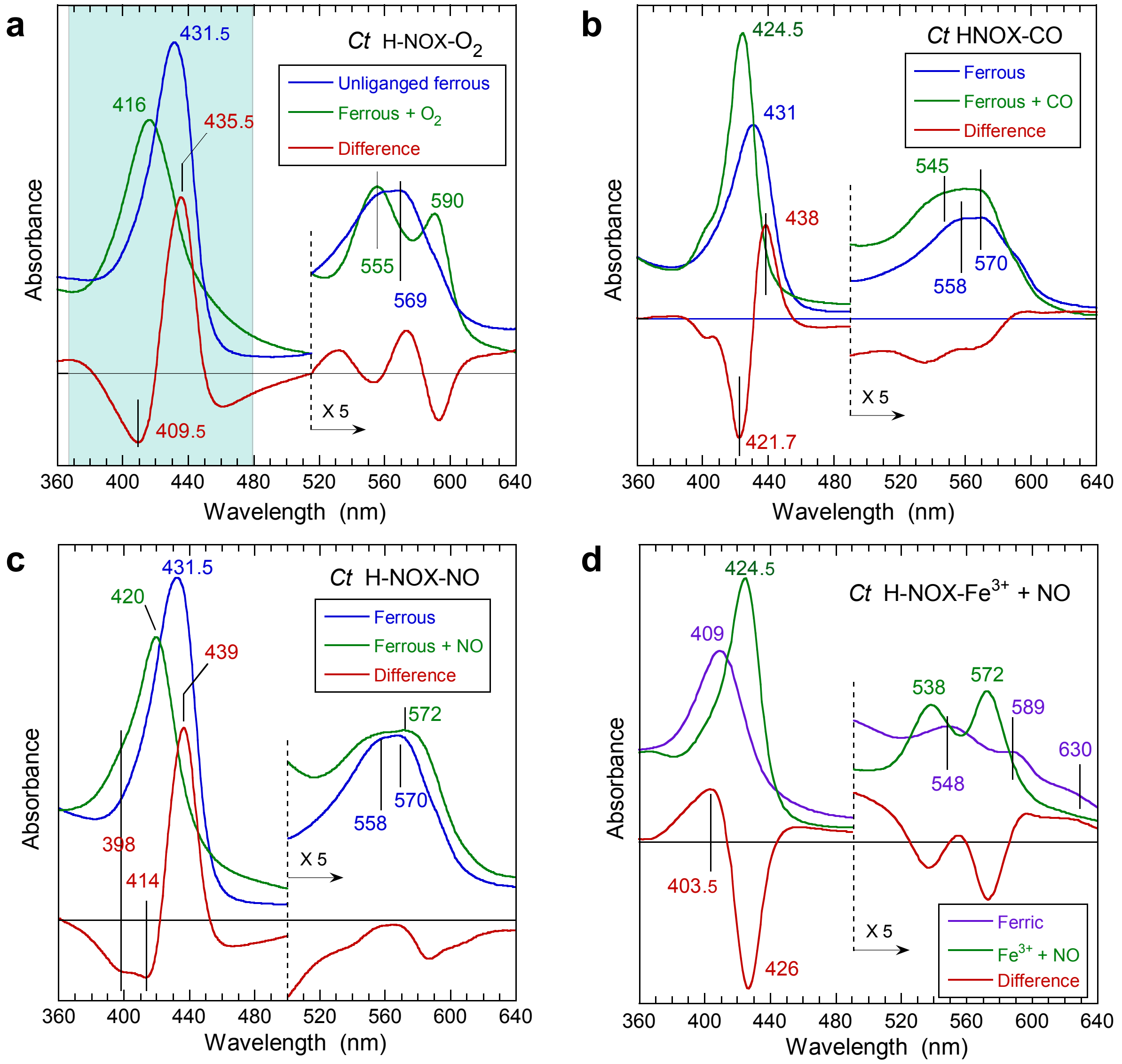

Supplementary Fig. S1. **Equilibrium absorption spectra of *Ct* H-NOX in various coordination states.** (**a**) Thawed *Ct* H-NOX (Fe^{2+} – O_2) and reduced (Fe^{2+}) species, together with the calculated difference. (**b**) NO-bounded (Fe^{2+} – CO). (**c** NO-bounded (Fe^{2+} – NO). (**d**) Oxidized form of *Ct* H-NOX (Fe^{3+}) and NO-bounded (Fe^{3+} – NO). See Experimental procedures for the preparation. Optical path length = 1 mm. In panel **a**, the blue portion of the spectrum represents the range probed by time-resolved absorption.

Supplementary Table S1. Peak positions in equilibrium absorption spectra of *Ct* H-NOX.

Proteins	Coordination	Soret band (nm)	Q-band (nm)
**Unliganded**			
sGC	Fe(II) 5c-His	431.5	555
sGC	Fe(III) 5c-His	393	550
Ct H-NOX	Fe(II) 5c-His	431 − 432	558 − 570
Ct H-NOX	Fe(III) 6c-His-(H_2O)	409	548 − 589
Ct H-NOX	Fe(III) 5c-His transient	390 − 400	−
**Heme-NO complex**			
sGC	Fe(II) 5c-NO	398	
Ct H-NOX	Fe(II) 6c-NO	420	550 − 572
	Fe(III) 6c-NO	424.5	538 − 572
**Heme-O_2 complex**			
Ct H-NOX	Fe(II) 6c-O_2	416	555 − 590
Mb	Fe(II) 6c-O_2	420	

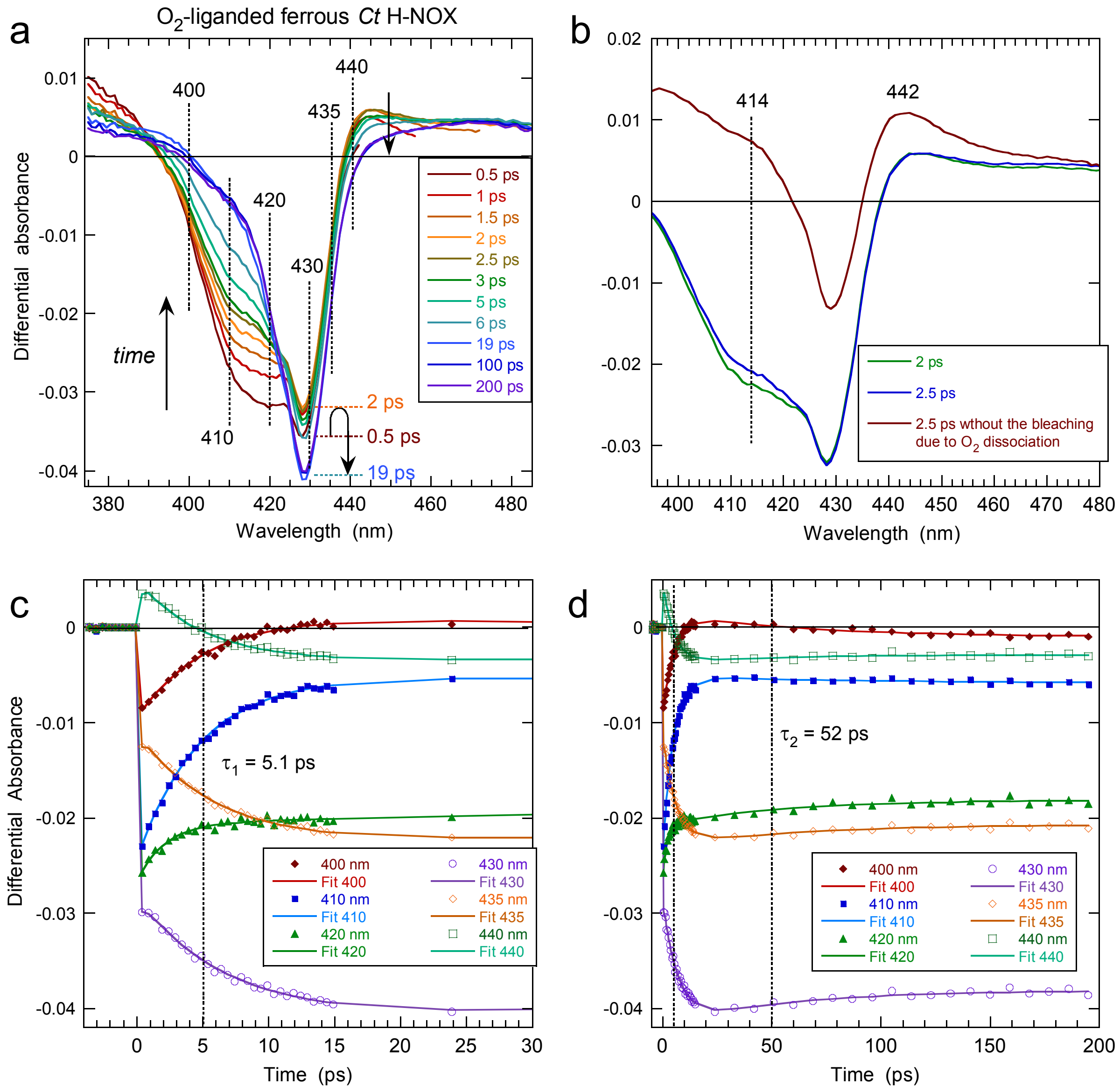

Supplementary Fig. S2. Dynamics in ferrous O_2-liganded *Ct* H-NOX. (**a**) Raw transient difference absorption spectra (spectra at positive delay *minus* spectrum at negative delay) after the photodissociation of O_2 from ferrous heme at increasing time delay. The wavelengths at which kinetics were analyzed (panels *c* and *d*) are indicated. (**b**) Spectrum at 2.5 ps from which the contribution of dissociated O_2 (bleaching) was removed by adding the equilibrium absorption spectrum of O_2-liganded *Ct* H-NOX (red). (**c** and **d**) Kinetics at particular wavelengths (see panel *a*) fitted to a sum of two exponentials with time constants in two time ranges (Supplementary Table S2).

Supplementary Table S2. Time constants of the fitted raw kinetics for photodissociated O_2−*Ct* H-NOX.

**Wavelength (a) (nm)**	**τ_1 (A_1) ps (%)**	**τ_2 (A_2) ps (%)**	**$A_{constant}$ (%)**
400	5.2 (*75*)	58 (*18*)	*7*
410	4.7 (*74*)	114 (*3*)	*23*
420	~1 (*22*)	54 (*10*)	*68*
430	6.7 (*24*)<br>~1 (*3*)	53 (*7*)	*66*
435	5.9 (*31*)<br>~1 (*12*)	60 (*6*)	*51*
440	5.2 (*50*)<br>~1 (*29*)	44 (*5*)	*16*

(a) These wavelengths do not correspond to the absorption maximum of the different species, but to wavelengths at which species can be probed efficiently in the transient spectra.

Supplementary result and discussion.

Excited states relaxation of unliganded *Ct* H-NOX sensor.

We recorded the heme excited states relaxation of ferrous unliganded *Ct* H-NOX (Supplementary Fig. S3). An instantaneous bleaching appears at 430.5 nm which decreases and is stabilized at 17 ps, centered at 428 nm, whereas the maximum of the induced absorption (449 nm) fastly shifts and decreases. Simultaneously, the isosbestic point shifts from 443 nm (at 1 ps) to 439 nm (at 7 ps), then shifts back to 445 nm after ~20 ps (Supplementary Fig. S3a), indicating the occurrence of at least two processes. We analyzed the time-wavelength data matrix using the Singular Value Decomposition method which yielded individual spectral components (Supplementary Fig. S3b). The associated kinetics (Supplementary Fig. S3c) comprise two fast exponential components ($\tau_1 = 2 \pm 0.1$ ps and $\tau_2 = 5.1 \pm 0.2$ ps). They are assigned to excited states decay and heme vibrational cooling, as similarly observed in other ferrous heme proteins excited either in the Q-bands[1,2,3] or in the Soret band[4,5] and are well established as the "classical route" for heme electronic relaxation.

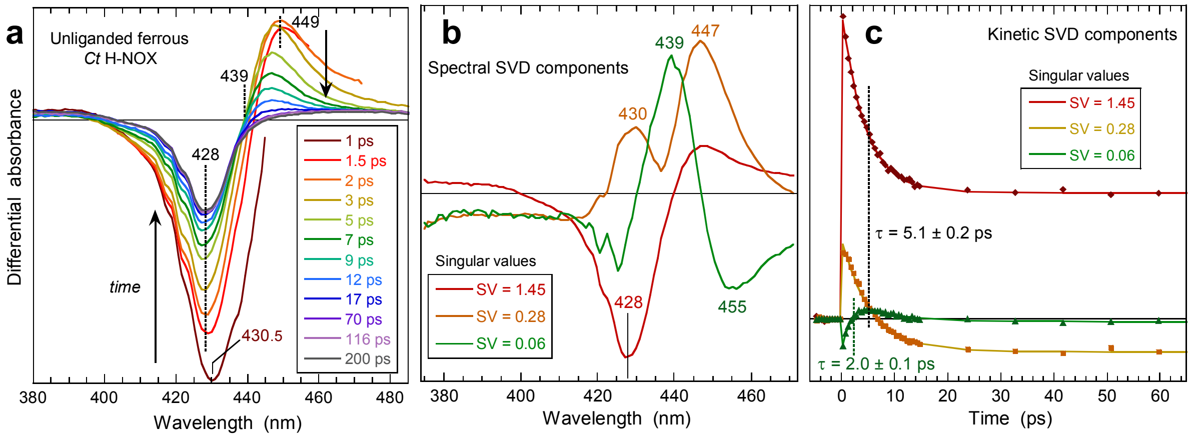

Supplementary Fig. S3. Excited states dynamics of ferrous unliganded *Ct* H-NOX. (**a**) Difference absorption spectra of the ferrous unliganded sensor at increasing time delay after photo-excitation at 564 nm. (**b**) Spectral components from Singular Value Decomposition of the time-wavelength data matrix. The relative absorbance is obtained by multiplying with the respective singular value. (**c**) SVD kinetic components with time constants of the fitted exponentials.

Importantly here, after the decrease of the induced absorption (440 – 460 nm) from excited states, the induced bleaching at 428 nm does not reach the original baseline. The electronic excited heme does not relax to the initial absorption of unliganded ferrous *Ct* H-NOX after 200 ps, even though the heme ground state is 5-coordinate (5-c) so that no ligand can be photodissociated. The spectral component SVD1 represents the formation of this final species which occurs simultaneously to the vibrational relaxation with time constant $\tau_2 = 5.1$

ps. Since the absorption decrease is centered at 428 nm, the hypothesis of photo-oxidation[6] should be considered for this process. However, no induced absorption band appears in the region 393 − 405 nm where the 5-c ferric heme absorbs, or at any other wavelengths, once the induced absorption vanished after 17 ps (Supplementary Fig. S3a) and photo-oxidation can be discarded as the mechanism.

The present result means that photo-excitation of the *Ct* H-NOX unliganded ferrous heme transiently changes its conformation but does not change its redox state, so that its absorption spectrum is close to that of the initial ferrous ground state, but with a lower absorption coefficient, which can originate from a change of orbitals overlap due to a change of heme distortion.

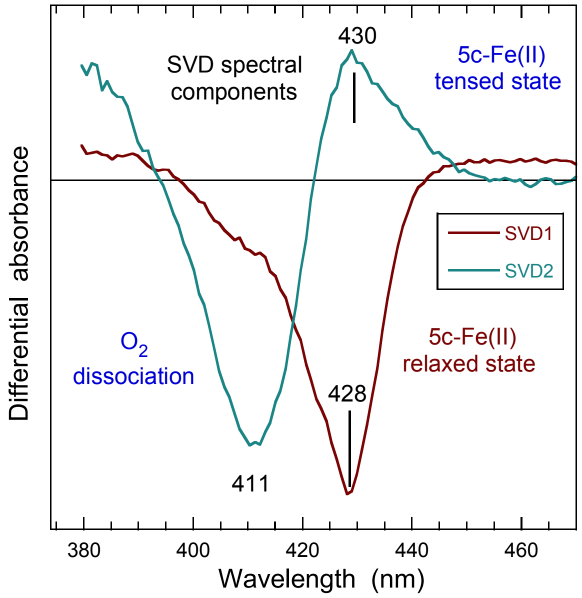

Supplementary Fig. S4. Spectral components from Singular Value Decomposition analysis of the time-wavelength data matrix of *Ct* H-NOX−O_2 measured to 5 ns.

Supplementary Table S3. Time constants of the $\mathbf{O_2}$ geminate rebinding for various heme proteins.

Protein	Function	τ_{G1} (A_1) ps (%)	τ_{G2} (A_2) ps (%)	$A_{constant}$ (a) (%)	Reference
Ct H-NOX	Sensor (putative O_2)	5.1 (*36*)	100 (*3*)	*61*	this work
L16A-Cyt c' (b)	NO transporter	7.5 (*27.5*)	120 (*22*)<br>2000 (*45.3*)	*5.2*	(7)
Myoglobin (c)	O_2 transporter	6.3 (*28.5*)	291 (*6*)	*65.5*	(8)
HbI (d)	H_2S transporter	6.0 (*20.5*)	396 (*18*)	*61.5*	(8)
HbII-III (d)	H_2S transporter	5.8 (*72*)	100 (5)	*23*	(8)
DosH (e)	O_2 Sensor	5.3 (*96*)	–	*4*	(9)
FixL (f)	O_2 Sensor	4.7 (*90*)	–	*10*	(9)
Af GcHK (g) globin-coupled histidine kinase	O_2 Sensor	–	2000 – 3000 (> *90*)	< *10*	(10)

(a) The constant term A_C quantifies the amount of O_2 which exits the protein and does not rebind geminately.
(b) From the bacterium *Alcaligenes xylosoxydans* Leu16Ala mutant. The wild type protein does not bind O_2.
(c) From horse heart.
(d) From the mollusc *Lucina pectinata*.
(e) From the bacterium *Escherichia coli*.
(f) From the bacterium *Bradyrhizobium japonicum*.
(g) From the bacterium *Anaeromyxobacter* sp.

Supplementary Table S4. Time constants of the **NO** geminate rebinding for various heme proteins.

**Protein**	**Function**	**τ_{G1} (A_1) ps (%)**	**τ_{G2} (A_2) ps (%)**	**$A_{constant}$ (a) (%)**	**Reference**
Ct H-NOX	Sensor (putative O_2)	6.8 (*78*)	200 (*4*)	*18*	this work
Cyt c' (b)	NO transporter	7.5 (*99*)	–	*1*	(11)
sGC (c)	NO receptor	7.5 (*97*)	–	*3*	(1)
Myoglobin (d) **Ferrous** heme	O_2 transporter	13 (*40*)	148 (*50*)	*10*	(8)
Myoglobin (d) **Ferric** heme	O_2 transporter	24 (*14*)	208 (*48*)	*38*	(8)
HbI (e)	H_2S transporter	8.0 (*36*)	90 (*62*)	*2*	(8)
HbII-III (e)	H_2S transporter	11 (*83*)	61 (*15*)	*2*	(8)
DosH (f)	O_2 Sensor	5.0 (*85*)	20 (*15*)	*0*	(12)
FixL (g)	O_2 Sensor	5.3 (*62*)	20 (*24*)<br>220 (*8*)	*6*	(9)
E. Coli YddV **Ferric** heme	O_2 Sensor	90 (*25*)	650 (*45*)	*30*	(13)

(a) The constant term A_C quantifies the amount of NO which exits the protein and does not rebind geminately.
(b) From *Alcaligenes xylosoxydans*.
(c) From bovine lung.
(d) From horse heart.
(e) From the mollusc *Lucina pectinata*.
(f) From the bacterium *Escherichia coli*.
(g) From the bacterium *Bradyrhizobium japonicum*.

Supplementary Table S5. Ratio of the singular values of SVD1 and SVD2 components associated with heme structural relaxation and ligands binding, respectively.

**Diatomics**	**SVD1 / SVD2**	**K_D (M)**
O_2	0.958 / 0.143<br>$\gamma = 6.7$	10^{-8}
NO	0.324 / 0.162<br>$\gamma = 2$	2.3×10^{-11}
CO	$\gamma < 1$	1.6×10^{-7}

For O_2 we used the value of k_{on} that we have measured here by time-resolved spectroscopy and the value of k_{off} in Wu *et al.*[14]

Supplementary references.

1. Negrerie, M., Bouzhir, L., Martin, J.-L. & Liebl, U. Control of nitric oxide dynamics by guanylate cyclase in its activated state. *J. Biol. Chem.* **276**, 46815−46821 (2001).
2. Ye, X. et al. Investigations of heme protein absorption line shapes, vibrational relaxation, and resonance Raman scattering on ultrafast time scales. *J. Phys. Chem. A* **107**, 8156−8165 (2003).
3. Negrerie, M., Cianetti, S., Vos, M. H., Martin, J.-L. & Kruglik, S. G. Ultrafast heme dynamics in ferrous versus ferric cytochrome *c* studied by time-resolved resonance Raman and transient absorption spectroscopy. *J. Phys. Chem. B* **110**, 12766−12781 (2006).
4. Wang, W. et al. Femtosecond multicolor pump−probe spectroscopy of ferrous cytochrome c. *J. Phys. Chem. B* **104**, 10789−10801 (2000).
5. Sun, Y. et al. Kinetic control of O_2 reactivity in H-NOX domains. *J. Phys. Chem. B* **120**, 5351−5358 (2016).
6. Chauvet, A. P., Agarwal, R., al Haddad, A., van Mourik, F. & Cramer, W. A. Photo-induced oxidation of the uniquely liganded heme *f* in the cytochrome b_6f complex of oxygenic photosynthesis. *Phys. Chem. Chem. Phys.* **18**, 12983−12991 (2016).
7. Andrew, C. R., Petrova, O. N., Lamarre, I., Lambry, J.-C., Rappaport, F. & Negrerie, M. The dynamics behind the affinity: controlling heme-gas affinity via geminate recombination and heme propionate conformation in the NO carrier cytochrome c′. *ACS Chem. Biol.* **11**, 3191−3201 (2016).
8. Ramos-Alvarez, C., Yoo, B.-K,. Pietri, R., Lamarre, I., Martin, J.-L., Lopez-Garriga, J. & Negrerie, M. Reactivity and dynamics of H_2S, NO, and O_2 interacting with hemoglobins from *Lucina pectinata*. *Biochemistry* **52**, 7007−7021 (2013).
9. Liebl, U., Bouzhir-Sima, L., Negrerie, M., Martin, J.-L. & Vos, M. H. Ultrafast ligand rebinding in the heme domain of the oxygen sensors FixL ans Dos : general regulatory implications for heme-based sensors. *Proc. Natl. Acad. Sci. USA.* **99**, 12771−12776 (2002).
10. Fojtikova, V., Stranava, M., Vos, M. H., Liebl, U., Hranicek, J., Kitanishi, K., Shimizu, T. & Martinkova, M. Kinetic analysis of a globin-coupled histidine kinase, *Af* GcHK: effects of the heme iron complex, response regulator, and metal cations on autophosphorylation activity. Biochemistry **54**, 5017−5029 (2015).
11. Kruglik, S. G., Lambry, J. C., Cianetti, S., Martin, J. L., Eady, R. R., Andrew, C. R. & Negrerie, M. Molecular basis for nitric oxide dynamics and affinity with *Alcaligenes xylosoxidans* cytochrome *c'*. *J. Biol. Chem*. **282**, 5053–5062 (2007).
12. Liebl, U., Bouzhir-Sima, L., Kiger, L., Marden, M. C., Lambry, J. C., Negrerie, M. & Vos, M. H. Ligand binding dynamics to the heme domain of the oxygen sensor Dos from *Escherichia coli*. *Biochemistry* **42**, 6527–6535 (2003).
13. Lambry, J.-C., Stranava, M., Lobato, L., Martinkova, M., Shimizu, T., Liebl, U. & Vos, M. H. Ultrafast spectroscopy evidence for picosecond ligand exchange at the binding site of a heme protein: heme-based sensor YddV. *J. Phys. Chem. Lett.* **7**, 69−74 (2016).
14. Wu, G., Liu, W., Berka, V., Tsai, A.-L. H-NOX from *Clostridium botulinum*, like H-NOX from *Thermoanaerobacter tengcongensis*, binds oxygen but with a less stable oxyferrous heme intermediate. *Biochemistry* **54**, 7098−7109 (2015).
